# Autologous Bone Marrow-Derived Stem Cells for Treating Diabetic Neuropathy in Metabolic Syndrome

**DOI:** 10.1155/2017/8945310

**Published:** 2017-10-01

**Authors:** Wei Liu, Fengchun Yu, Zhenghong Zhou, Yi-Chen Li, Dongsheng Fan, Kai Zhu

**Affiliations:** ^1^Department of Neurology, Beijing Haidian Hospital, Beijing 100080, China; ^2^Division of Engineering in Medicine, Brigham and Women's Hospital, Harvard Medical School, Cambridge, MA 02139, USA; ^3^Harvard-MIT Division of Health Sciences and Technology, Massachusetts Institute of Technology, Cambridge, MA 02139, USA; ^4^Department of Neurology, Peking University Third Hospital, Beijing 100191, China; ^5^Department of Cardiac Surgery, Zhongshan Hospital, Fudan University, Shanghai 200032, China; ^6^Shanghai Institute of Cardiovascular Disease, Shanghai 200032, China

## Abstract

Diabetic neuropathy is one of the most common and serious complications of diabetes mellitus and metabolic syndrome. The current therapy strategies, including glucose control and pain management, are not effective for most patients. Growing evidence suggests that infiltration of inflammation factors and deficiency of local neurotrophic and angiogenic factors contribute significantly to the pathologies of diabetic neuropathy. Experimental and clinical studies have shown that bone marrow-derived stem cells (BMCs) therapy represents a novel and promising strategy for tissue repair through paracrine secretion of multiple cytokines, which has a potential to inhibit inflammation and promote angiogenesis and neurotrophy in diabetic neuropathy. In this review, we discuss the clinical practice in diabetic neuropathy and the therapeutic effect of BMC. We subsequently illustrate the functional impairment of autologous BMCs due to the interrupted bone marrow niche in diabetic neuropathy. We anticipate that the functional restoration of BMCs could improve their therapeutic effect and enable their wide applications in diabetic neuropathy.

## 1. Introduction

Diabetic neuropathy is one of the most frequent complications in diabetes mellitus and metabolic syndromes, which cause high rate of disability and mortality [1, 2]. Diabetic neuropathy can affect a wide spectrum of peripheral nerves, including pain fibres and motoneuron and autonomic nervous system [3]. However, its pathophysiology is not yet fully elucidated. The treatment of hyperglycemia is a recommended strategy to reduce the incidence of diabetes neuropathy [4]. However, it showed a marginal effect on preventing diabetic neuropathy in type 2 diabetes mellitus, indicating that other factors might be also involved in the nerve injury in these patients [4]. The other conventional therapeutics, such as aldose reductase inhibitor or *α*-lipoic acid, also showed limited therapy response in the progressive stage of diabetes neuropathy [5]. Recently, it is speculated that diabetic neuropathy is secondary to the deficiency of local growth factors, besides which, a number of inflammatory mediators, such as interleukins and chemokines, are involved in the progression of diabetic neuropathy [6]. There is a growing interest in the scientific community for cellular therapies, such as utilizing of BMCs, for the treatment of diabetes and its complications [7]. In the past decade, BMCs have been applied as a promising therapy for diabetic neuropathy because of their multipotency and their comprehensive paracrine secretion of anti-inflammatory cytokines, proangiogenic factors, and neurotrophic factors [8]. However, diabetes neuropathy may jeopardize the physiological function of autologous BMCs, yielding their modest therapy efficacy [9]. Here, we review the clinical profile of diabetic neuropathy and BMC therapy. Finally, some solutions to restore BMC function in diabetes mellitus are addressed.

## 2. Current Clinical Management of Diabetic Neuropathy

Diabetic neuropathy has been defined as the presence of symptoms and/or signs of peripheral nerve dysfunction in people with diabetes after the exclusion of other causes, which is the most common complication of diabetes, affecting 30–50% of individuals with diabetes mellitus. Patients with diabetic neuropathy have poor quality of life and reduced work productivity, along with high healthcare costs. Cardiac autonomic neuropathy has been hailed as the “Prophet of Doom” and accounts for 20–34% of all patients with DM [10]. It is conducive to increased risk of myocardial infarction, malignant arrhythmia, and sudden death.

**Figure 1:**
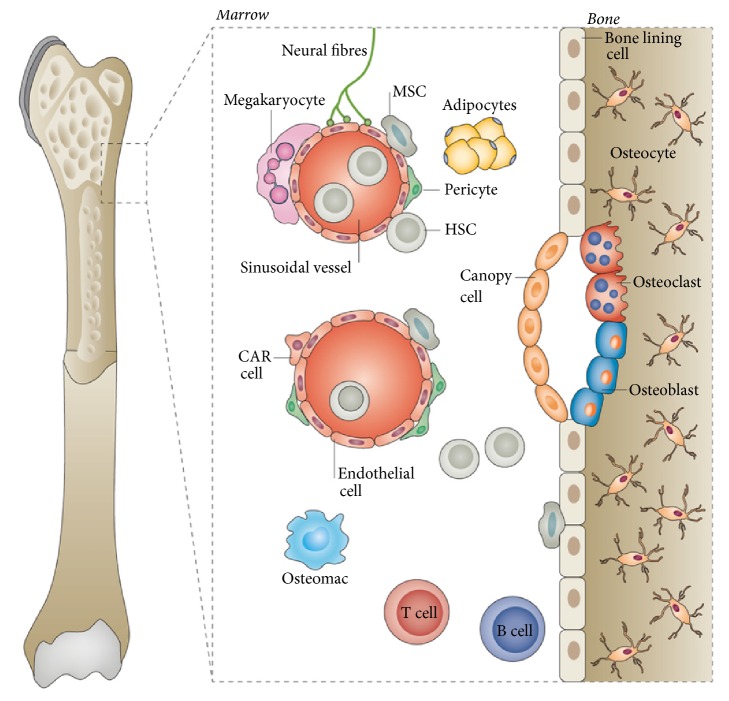
*Schematic of bone marrow niche*. Bone marrow niche is anatomical compartments constituted by cellular and extracellular components that mediate stem cell maintenance and expansion. Adapted by permission from [45] copyright Nature Publishing Group 2016.

Diabetic neuropathy has been known for over two decades. The pathological mechanisms have been shown to be complicated (Figure 1). Diabetes mellitus often cause several prolonged metabolic disorders, including hyperglycemia, hyperinsulinemia, growth factor abnormalities, and dyslipidemia. These disorders could bring a complicated alternation, such as alteration of polyol metabolism and excessive oxidative stress, which could be triggered for the potential antigenic leakage and damage in nerve fibres. As a result, organic and structural nerve damage could occur. However, the regeneration capacity in nerve is limited, causing the irreversible neuropathy in patients with diabetes mellitus. Several therapeutics, such as aldose reductase inhibitor and *α*-lipoic acid, have been developed based on these mechanisms. For instance, data from clinical trials showed that aldose reductase could improve the conduction velocity of motor and sensory nerves [11]. Nevertheless, the other involved mechanisms, such as nonenzymatic glucosylation, could be also targeted to develop novel therapeutics in future.

The high quality clinical evidence from 17 randomized studies (7 in type 1 diabetes mellitus, 8 in type 2 diabetes mellitus, and 2 in both types) showed that strict glucose control significantly prevented the development of diabetic neuropathy in patients with type 1 diabetes mellitus [12]. In contrast, the strict glucose control could reduce the incidence of neuropathy in type 2 diabetes mellitus, but without statistical significance (*P* = 0.06). However, the benefit from the aggressive glucose control has to be balanced against the increased risk of low blood glucose level, which could be dangerous and may lead to brain injury.

Diabetes mellitus also could lead to the autoimmune abnormality, which is a major cause of inflammation in the nerves and subsequent nerve damage. In one recent study, researchers found that blood serum autoimmune antibodies were significantly present in the patients with peripheral diabetic neuropathy [13]. Microvascular insufficiency in diabetes mellitus may also contribute to the pathogenetic mechanism in nerve neuropathy. The adequate blood supply from microvessels is crucial to maintain the normal structure and function in nerves. The strict blood glucose control is applied in patients with diabetes mellitus [4]. Poor glycemic control is clearly associated with the development of diabetic neuropathy. In addition, it has been found that diabetes could reduce neurotrophic factors in peripheral nerves by reducing the anterograde and retrograde axonal transport, thus making significant influence on neural morphology and conductivity. A meta-analysis including ten studies showed aldose reductase inhibitor improved cardiac autonomic function, with an acceptable safety profile for all agents except tolrestat [14].

## 3. Characteristics of BMCs

BMCs constitute a heterogeneous cell population, mainly including hematopoietic stem cells (HSCs), mesenchymal stem cells (MSCs), and endothelial progenitor cells (EPCs) [15]. In response to tissue injury or exogenously by cytokine stimulation, the endogenous BMCs can be mobilized to conduct their repair function [16]. The isolated BMCs can be cultured and expanded for implantation therapy [17–23]. Autologous BMCs are being explored in early clinical trials as therapy for various health disorders owing to no requirement for systemic immunosuppression or donor matching [24]. Collectively, the mobilized, intravascularly or locally delivered BMSCs can home into the damaged tissues or organs.

HSCs give rise to both the myeloid and lymphoid lineages of blood cells [25, 26]. However, HSCs are also a large pool of cell population. The identification of HSCs is usually dependent on the specific cell surface markers, such as CD34 and CD133. For instance, CD34+ HSCs have been widely used in clinical trials to reconstitute the deficient hematopoietic system after radiation or chemotherapy [25]. Besides that, it has also been found that CD34+ HSCs could promote therapeutic angiogenesis in tissue ischemia models [27]. One of the underlying mechanisms is their paracrine secretion of angiogenic growth factors that induce microvasculature [28].

Mesenchymal stem cells (MSCs) can be isolated from bone marrow as well as in several other adult tissues [29]. They are highly proliferative and have capacity to differentiate into various cell types, thus making MSCs a promising candidate for a wide range of medicine applications. In recent studies, it has been found that implantation of allogeneic MSCs was protected from immune detection through “hit and run” (immune evasive) instead of hypoimmunogenic or immune privileged mechanisms [30]. In addition, the isolated MSCs could also be transfected with various therapeutic genes to exert improved functions after implantation [31].

EPCs are a subset of myeloid/monocyte cells whose surface can express endothelial markers, such as kinase insert domain receptor (KDR), CD31, CD133, or von Willebrand factor (vWf) [32]. Studies suggest that EPCs have favorable survival and a better response toward angiogenic growth factors compared with mature endothelial cells. The circulating EPCs concentration has been used for biomarkers for some disease detection and staging [33]. In addition, it has been demonstrated that EPCs have the benefit of proangiogenesis in ischemic tissues mainly via paracrine secretion rather than differentiation into vascular cells [34].

## 4. BMC Therapy for Diabetic Neuropathy

It has been found that growth factor therapy is an attractive option for diabetic neuropathy [35]. The administration of growth factors can promote angiogenesis and neural regeneration. Some growth factors, known as angioneurins, have both angiogenic and neurotrophic properties. In one study, researchers injected vascular endothelial growth factor (VEGF) encoding plasmids into diabetic animal models. The therapeutic showed enhancement in angiogenesis of vasa nervorum, increase in nerve fibre density, and normalization in nerve conductivity velocity [35]. A randomized, double-blinded trial in humans showed that VEGF could evoke statistically significant symptomatic improvement of diabetic neuropathy [36]. Other growth factors, such as insulin-like growth factor (IGF), nerve growth factor (NGF), and ciliary neurotrophic factor, were also investigated in experimental animals with diabetic neuropathy [37]. They were found to ameliorate development of neuropathy and improve nerve function. Therefore, a therapeutic strategy to promote angiogenesis and neuron regeneration may be promising in treatment of diabetic neuropathy.

In this context, BMCs, which can produce multiple angiogenic and neurotrophic growth factors and potentially supplement specific type of cells required for vascular or neuron regeneration, have advantages over growth factor therapy [38]. Currently, preclinical studies showed their potential therapeutic effects in diabetic neuropathy. One of the advantages to use BMCs is the possibility of autograft; that is, they can be harvested from a patient and autologously administered back. In rat models with type 1 diabetes, bone marrow-derived MSCs were found to ameliorate hypoalgesia and normalize nerve conduction velocities [39]. Although MSCs have been proved to support de novo regeneration of neuronal cells, paracrine properties seem to be more prevalent. Besides neurotrophic and angiogenic factors such as NGF, VEGF, and IGF-1, MSCs can also produce anti-inflammatory cytokines that moderate leukocyte recruitment in injured nerve [40]. This multiple-aspected action on inflammation, nerves, and vessels is also exerted by EPCs. In a type 1 diabetes mouse model, EPC administration increased the level of VEGF, basic fibroblast growth factor, and glioma-associated oncogene family zinc finger 1 protein, thus booming the proliferation of Schwann and endothelial cells [41]. Besides that, the endogenous cell mobilization is an alternative to cell transplantation therapy. In a rodent model with diabetic neuropathy, intraperitoneal administration of granulocyte colony-stimulating factor (G-CSF) recruited stem cells from bone marrow to improve nerve function [42]. However, no proof of fusion with local cells or neuron cyte differentiation was observed, thus indicating that a paracrine action was conducted by the recruited stem cells.

## 5. Functional Impairment of BMC in Diabetic Neuropathy

The reservoir of BMCs, namely, bone marrow niches, are anatomical compartments constituted by cellular and extracellular components. They are crucial for the stem cell maintenance and expansion (Figure 1) [43–45]. As functional entity with a high plasticity capacity, bone marrow niche is able to response rapidly for the signals from the body. Under aging and disease conditions, bone marrow niches could undergo extensive remodeling, thus altering the properties of resident BMCs. Interruption in bone marrow niche homeostasis may lead to abnormalities of stem cell renewal, lineage specification, and mobilization.

Sympathetic and nociceptive fibres stimulate the release of BMCs into bloodstream [46]. Recent report demonstrated that the occurrence of diabetic autonomic neuropathy might result in the defective mobilization of BMSCs [47]. In a clinical trial (NCT01102699), patients with diabetes mellitus showed impaired mobilization ability of HSCs and EPCs while being in response to G-CSF [48]. In another study, Albiero et al. demonstrated that both experimental murine models of type 1 and type 2 diabetes develop bone marrow autonomic neuropathy with an impaired mobilization of BMCs by upregulation of src homology and collagen homology domain and downregulation of Sirtuin 1 (Sirt1) [49]. Mobilization of vascular stem cells plays an important role in repairing tissue damage by promoting angiogenesis and reperfusion. Therefore, impaired mobilization of cells is expected to promote cardiovascular disease related to cardiac autonomic neuropathy. Collectively, these reports indicate that diabetic neuropathy may adversely affect stem cell-based therapies in patients with diabetic neuropathy.

## 6. Functional Restoration of BMCs

To improve BMC mobilization in diabetes, some types of cytokines could be alternative to G-CSF. In a previous study, researchers found that G-CSF failed to mobilize bone marrow stem cells in diabetic patients. However, when G-CSF was administrated in conjunction with plerixafor, an antagonist of the SDF-1/CXCR4 axis, the mobilization ability of stem cells was observed to be restored [48, 50].

For the ex vivo isolated BMSCs, genetic engineering could be an efficient strategy to rescue functionality of stem cells. It has been well known that cell development and function could be regulated through the modulation of miRNA level, thus enabling miRNA as the therapeutic target in BMCs [51, 52]. For instance, downregulation of microRNA-155 in hematopoietic stem cells was found to maintain the pool of original stem cells in diabetic bone marrow [53]. In addition, downregulation of microRNA-15a and microRNA-16 could ameliorate angiogenic potential of diabetic EPCs [54].

Much evidence showed that function of BMCs from diabetes could be restored through administration of some gaseous signal molecule, such as hydrogen sulfide. In one study from Liu et al., hydrogen sulfide was found to restore the angiogenic functions of EPCs by upregulating expression of Ang-1 [55]. In a recent study, Cheng et al. demonstrated that decreased cystathionine *γ*-lyase-mediated hydrogen sulfide bioavailability is the potential reason of impairment of BMC function in diabetes [56]. They applied the hydrogen sulfide donor treatment or overexpression of CSE gene in diabetic BMCs and found improved repair capacity in diabetic critical limb ischemia.

Other strategies include adding of the culture media with a supplement of cytokines [57, 58]. For instance, Kim et al. reported that Krüppel-like factor 2 (KLF2), a critical angiogenic factor, was dramatically decreased in diabetic bone marrow-derived MSCs [59]. The addition of oxytocin into the culture medium could significantly restore function of MSCs through upregulation of KLF-2 expression.

## 7. Perspective and Conclusions

As evidence grows on the safety of autologous BMC therapy in a number of clinical trials, the potential to translate this therapeutic strategy into a pilot clinical trial for diabetic neuropathy is very promising. However, several steps still need to be completed before that can be a reality. Firstly, our current knowledge of restoring the proper bone marrow niche microenvironment in patients with diabetes requires more investigation. The modulation of bone marrow niche is vital to obtain the healthy candidate cells. In addition, the isolated BMCs need to be functionally modified through genetic engineering and other strategies. In conclusion, BMC therapy may become a potential therapeutic option to treat diabetic neuropathy.
